# Backpack satellite transmitters reduce survival but not nesting propensity or success of greater sage‐grouse

**DOI:** 10.1002/ece3.10820

**Published:** 2023-12-18

**Authors:** Bryan S. Stevens, Courtney J. Conway, Cody A. Tisdale, Kylie N. Denny, Andrew Meyers, Paul Makela

**Affiliations:** ^1^ Idaho Cooperative Fish and Wildlife Research Unit, Department of Fish and Wildlife Sciences University of Idaho Moscow Idaho USA; ^2^ U.S. Geological Survey, Idaho Cooperative Fish and Wildlife Research Unit University of Idaho Moscow Idaho USA; ^3^ U.S. Bureau of Land Management Boise Idaho USA; ^4^ Present address: Pacific Northwest Research Station U.S. Forest Service La Grande Oregon USA; ^5^ Present address: School of the Environment Washington State University Pullman Washington USA; ^6^ Present address: Oregon Department of Fish and Wildlife The Dalles Oregon USA

**Keywords:** *Centrocercus urophasianus*, GPS transmitter, Idaho, nest survival, radio telemetry, sagebrush steppe, VHF transmitter

## Abstract

Telemetry technology is ubiquitous for studying the behavior and demography of wildlife, including the use of traditional very high frequency (VHF) radio telemetry and more recent methods that record animal locations using global positioning systems (GPS). Satellite‐based GPS telemetry allows researchers to collect high spatial–temporal resolution data remotely but may also come with additional costs. For example, recent studies from the southern Great Basin suggested GPS transmitters attached via backpacks may reduce the survival of greater sage‐grouse (*Centrocercus urophasianus*) relative to VHF transmitters attached via collars that have been in use for decades. While some evidence suggests GPS backpacks reduce survival, no studies have examined the effects of GPS backpacks on breeding behavior and success. Therefore, we compared survival, breeding behavior, and nest success of sage‐grouse hens marked with both VHF collars and GPS backpack transmitter over a 7‐year period in central Idaho, USA. GPS backpacks reduced spring–summer survival of sage‐grouse hens relative to hens with VHF collars, where daily mortality probability was 68%–82% higher from March 1 to August 1. Yet satellite GPS backpacks did not consistently affect nest success or the likelihood or timing of nest initiation relative to VHF collars. Daily nest survival varied annually and with timing of nest initiation and nest age, but marginal effects of transmitter type were statistically insignificant and interactions between transmitter type and study year produced no meaningful patterns. Our results corroborate recent studies for the effect of satellite GPS backpacks on sage‐grouse survival, but also suggest that these transmitters do not appear to affect components of fecundity. Our results therefore add important context to recent debate surrounding the effects of GPS backpacks on sage‐grouse, and the relative strengths and weaknesses of different transmitter types for understanding behavior and population dynamics.

## Graphical abstract

We studied survival, breeding behavior, and nest success of sage‐grouse hens marked with very high frequency (VHF) and satellite global positioning systems (GPS) transmitters over a 7‐year period in central Idaho, USA. We found that satellite GPS transmitters reduced spring–summer survival of sage‐grouse hens relative to VHF‐marked hens, where probability of survival from March 1 to August 1 was 17% higher for sage‐grouse hens marked with VHF transmitters. However, nesting propensity and timing of initiation were indistinguishable between hens marked with satellite GPS transmitters and those marked with VHF transmitters.

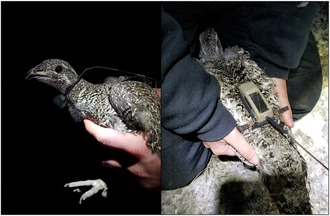

## 1 INTRODUCTION

Documenting behavior and estimating demographic parameters are common goals for research, monitoring, and management of animal populations, where behavioral and demographic data play pivotal roles for understanding many facets of animal ecology. Radio telemetry is a ubiquitous technology for studying animal behavior and demography, and the use of this technology has increased in recent decades. Development of very high frequency (VHF) radio telemetry allowed for tracking of individual animals with minimal disturbance by researchers (Millspaugh et al., 2012; Millspaugh & Marzluff, 2001) and facilitated inferences about a broad array of ecological processes, including breeding behavior, migration, habitat use, and survival (Aldridge & Brigham, 2002; Beck et al., 2006; Blomberg et al., 2013; Connelly et al., 1993; Dahlgren et al., 2010; Fedy et al., 2012; Fischer et al., 1993; Leonard et al., 2000). Telemetry data and the models built from those data are therefore commonly used to inform conservation and management of populations and habitats for sensitive species (Aldridge & Boyce, 2007; Fedy et al., 2014). More recently, the development of techniques based on global positioning systems (GPS) that collect animal location data remotely via satellites provided a paradigm shift in the collection of data with fine spatial and temporal resolution, and enormous volumes of location data followed (Cagnacci et al., 2010). High‐resolution data from satellite GPS transmitters have provided unique insights into ecological phenomena that are expressed over both very fine (e.g., lek attendance; Wann et al., 2019) and very large scales (e.g., migration; Newton et al., 2017), and thus the use of GPS‐based telemetry has become commonplace in wildlife ecology.

In spite of the common use of telemetry technologies, the effects of attaching transmitters and monitoring radiomarked animals are not always benign. Transmitters may negatively affect many aspects of a species life history, including their behavior, energetics, body condition, and demography (Barron et al., 2010). For example, transmitters reduced reproductive effort (e.g., nest initiation rates, time spent incubating eggs; Paquette et al., 1997; Rotella et al., 1993) and body mass (Greenwood & Sargeant, 1973; Kesler et al., 2014) of mallards (*Anas platyrhynchos*), and have decreased reproductive success (e.g., nest success, chick survival; Caizergues & Ellison, 1998; Erikstad, 1979; Warner & Etter, 1983) and survival (Marks & Marks, 1987; Small & Rusch, 1985) of Galliformes. Transmitter weights <3% of body mass are commonly deployed, yet the implications of transmitter weights for behavior and demography of birds appear highly variable (Barron et al., 2010). Studies of the effects transmitters on Galliformes have also provided inconsistent results and include reports of no transmitter effects (e.g., Hines & Zwickel, 1985). Moreover, the effects of transmitters may vary among populations or individuals (Lance & Watson, 1978; Small & Rusch, 1985) and attributes of transmitter design and deployment methods may alter their effects on monitored species. For example, transmitter positioning, attachment method (e.g., necklace vs. backpack), and weight (either absolute or relative to body mass) have all affected the behavior and demography of Galliformes (Burger et al., 1991; Severson et al., 2019; Small & Rusch, 1985; Warner & Etter, 1983).

Recent studies suggested negative effects of rump‐mounted, backpack‐style transmitters on survival and physical condition of greater sage‐grouse (*Centrocercus urophasianus*; sage‐grouse; Kircher et al., 2020; Severson et al., 2019), a sagebrush obligate that is often viewed as an umbrella species for the conservation of sagebrush ecosystems (Rowland et al., 2006). Necklace‐style VHF transmitters have been used to study grouse for >40 years (Amstrup, 1980) and are commonly used on sage‐grouse (Connelly et al., 2003), whereas backpack‐style satellite GPS transmitters have become available more recently but are now commonly employed (e.g., Kircher et al., 2020; Severson et al., 2019). Satellite GPS transmitters are attached via backpacks (rather than via collars) because they require solar panels exposed to sunlight to keep charged, and they increase the frequency and resolution of location data and reduce field monitoring efforts relative to VHF transmitters. However, they may come at the cost of reduced survival. Sage‐grouse with satellite GPS transmitters attached via backpack harnesses had lower annual and season‐specific survival than those with VHF collars in Nevada and California (Severson et al., 2019). Survival estimates for adult sage‐grouse marked with backpack GPS transmitters were also lower relative to those marked with VHF collars in Oregon (Foster et al., 2018), but those estimates were imprecise due to small sample sizes. The exact mechanisms by which backpack‐style GPS transmitters negatively impact demography of sage‐grouse relative to VHF collars are unclear, but hypotheses relate to increased transmitter weight, the backpack deployment design and attachment harness, and increased visibility resulting from reflective solar panels that are needed to reduce battery weight (Kircher et al., 2020; Severson et al., 2019).

Several studies provided evidence that backpack‐style GPS transmitters may reduce survival of sage‐grouse, but none examined the effects of backpack GPS transmitters on nesting behavior and success. Backpack‐style transmitters have negatively affected nest initiation and incubation behaviors of other species (e.g., waterfowl; Paquette et al., 1997; Rotella et al., 1993), and thus the hypothesis that deployment design and attachment methods drive changes in transmitter effects leads to predictions that backpack GPS transmitters typically used by sage‐grouse researchers could negatively impact survival, nesting behavior, and nest success of female sage‐grouse. The transmitter weight hypothesis predicts that heavier weight of GPS transmitters should decrease survival, but also predicts negative impacts to reproduction (e.g., decreased nesting propensity and success) if increased transmitter weight is detrimental to hen body condition or mobility during the nesting season. The hypothesis that solar panel reflectance increases visibility of GPS transmitters to avian predators (Burger et al., 1991) also suggests that backpack GPS transmitters would increase visibility of nesting hens to avian nest predators (e.g., common ravens; *Corvus corax*), and would therefore lead to predictions of reduced nest success and reduced survival by nesting hens.

While many hypotheses predict adverse effects of GPS transmitters on breeding sage‐grouse relative to VHF marked birds, this is not uniformly true. For example, nesting sage‐grouse can be monitored remotely using GPS transmitters, and hence the need for on‐the‐ground visits to nest sites is reduced. Increased depredation by avian predators is a common cause of nest failure after visitation by researchers (Götmark, 1992), as is sage‐grouse nest abandonment after disturbance by researchers (e.g., flushing of hens off their nest; Gibson et al., 2015). Therefore, we propose an additional hypothesis that differences in nest success will be driven by differences in observer disturbance between transmitter types. This hypothesis leads to a directionally different prediction because reduced observer disturbance afforded by GPS transmitters would result in higher nest success (relative to grouse marked with VHF collars). Nonetheless, effects of transmitter type (GPS vs. VHF) on sage‐grouse nest initiation and nest success have not been tested. Consequently, the objectives of this study were to test for effects of satellite GPS transmitters attached via backpacks (relative to VHF transmitters attached via collars) on: (1) survival of female sage‐grouse during the spring–summer period, (2) sage‐grouse nesting behavior (i.e., initiation and timing), and (3) sage‐grouse nest survival. Thus, our goals were to determine whether sage‐grouse marked with backpack‐style GPS transmitters have lower survival (as reported in the southern portion of their range), to assess for the first time the potential for backpack GPS transmitters to modify sage‐grouse nesting behavior and reproductive success, and to evaluate the observed patterns relative to expectations proposed under the hypotheses outlined above.

## 2 MATERIALS AND METHODS

### 2.1 Study area

**FIGURE 1:**
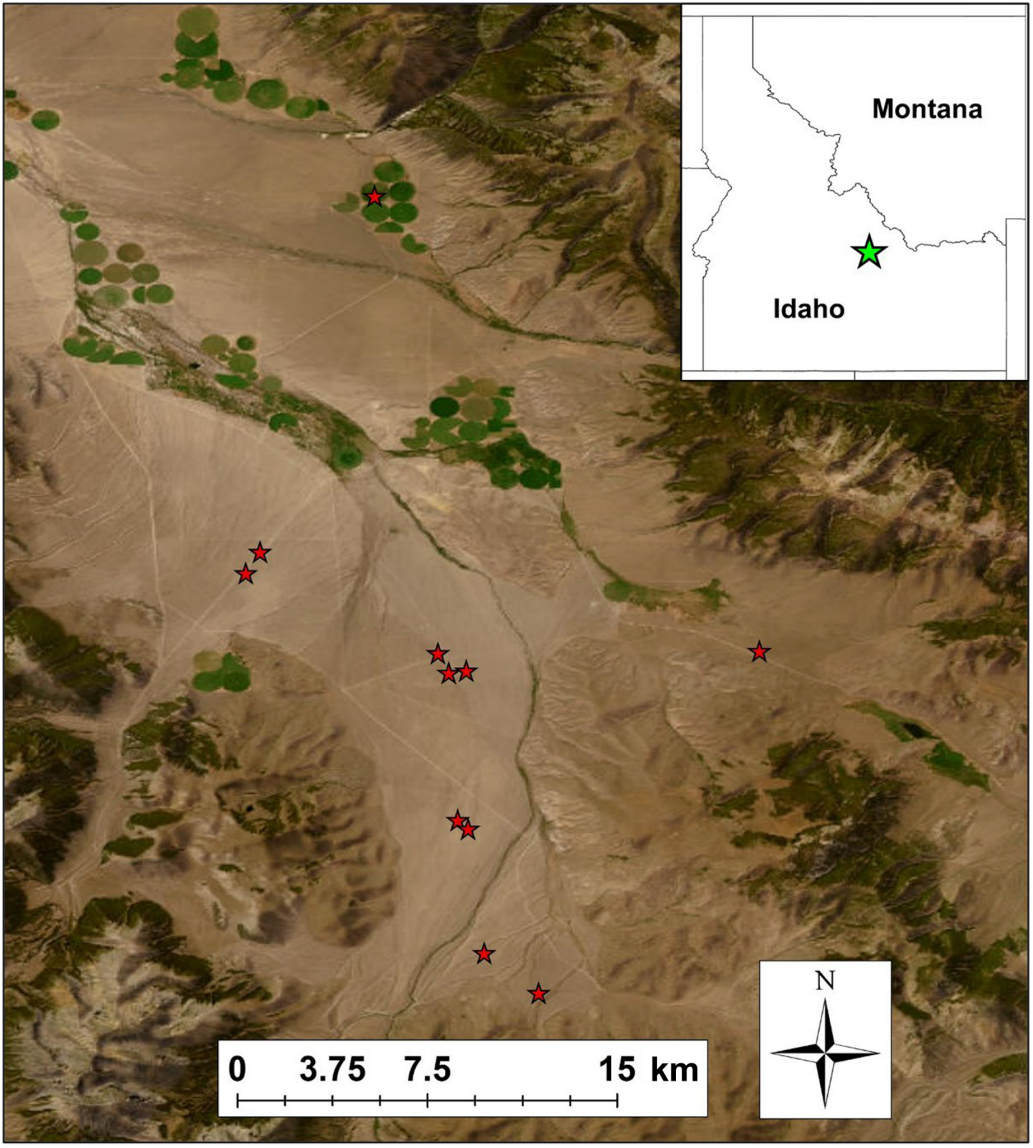
Map of the Pahsimeroi Valley study area in central Idaho, USA, where we assessed transmitter effects on female survival, nesting behavior, and nest survival (2016–2022). Red stars on the satellite image of the study area represent sage‐grouse leks. The green star on the statewide map (inset) is the approximate location of the Pahsimeroi Valley study site.

We studied survival and nest success of sage‐grouse during the breeding season from 2016 to 2022 in the Pahsimeroi Valley of central Idaho, which lies in the north central portion of the geographic range of sage‐grouse (Schroeder et al., 2004). The study area was located approximately 45‐km southeast of Challis, Idaho, at the upper end of the Pahsimeroi River (Figure 1) between the Lemhi Range to the northeast and the Lost River Range to the southwest. The site consisted of relatively flat topography in most locations but included sloping foothills and ridges at higher elevations. The Pahsimeroi River bisects the valley, as do several small creeks and irrigation ditches. Soils were moderately deep (>150 cm) and composed of gravely loam and cobble with 1%–40% slopes. Elevations across the study area ranged from 1796 to 2454 m ($\bar{x}$ = 1999 m; USGS National Elevation Dataset), and precipitation is dominated by snow that falls from November to March. The 30‐year normal precipitation averages 220 mm per year, and a monthly average temperature ranged from a low of −8.2°C (Dec) to a high of 18.6°C (Jul) based on a recent 30‐year normal (1980–2010). The plant community was dominated by low sagebrush (*Artemisia arbuscula*), with Wyoming big sagebrush (*A. tridentata wyomingensis*) and three‐tip sagebrush (*A. tripartata*) on the deeper toe slopes and ridges. Dominant perennial grasses included Sandberg bluegrass (*Poa secunda*), bluebunch wheatgrass (*Pseudoroegneria spicata*), Idaho fescue (*Festuca idahoensis*), and Thurber's needlegrass (*Achnatherum thurberianum*). Landownership in the valley is a combination of US Bureau of Land Management, Idaho State Trust, and privately owned lands. Dominant land uses in the area have included mining and livestock grazing (domestic cattle and sheep), where livestock grazing by cattle remains the dominant use. Land use at the north end of the valley included some center‐pivot irrigated agriculture, with alfalfa, hay, and wheat as primary crops.

### 2.2 Field methods

**FIGURE 2:**
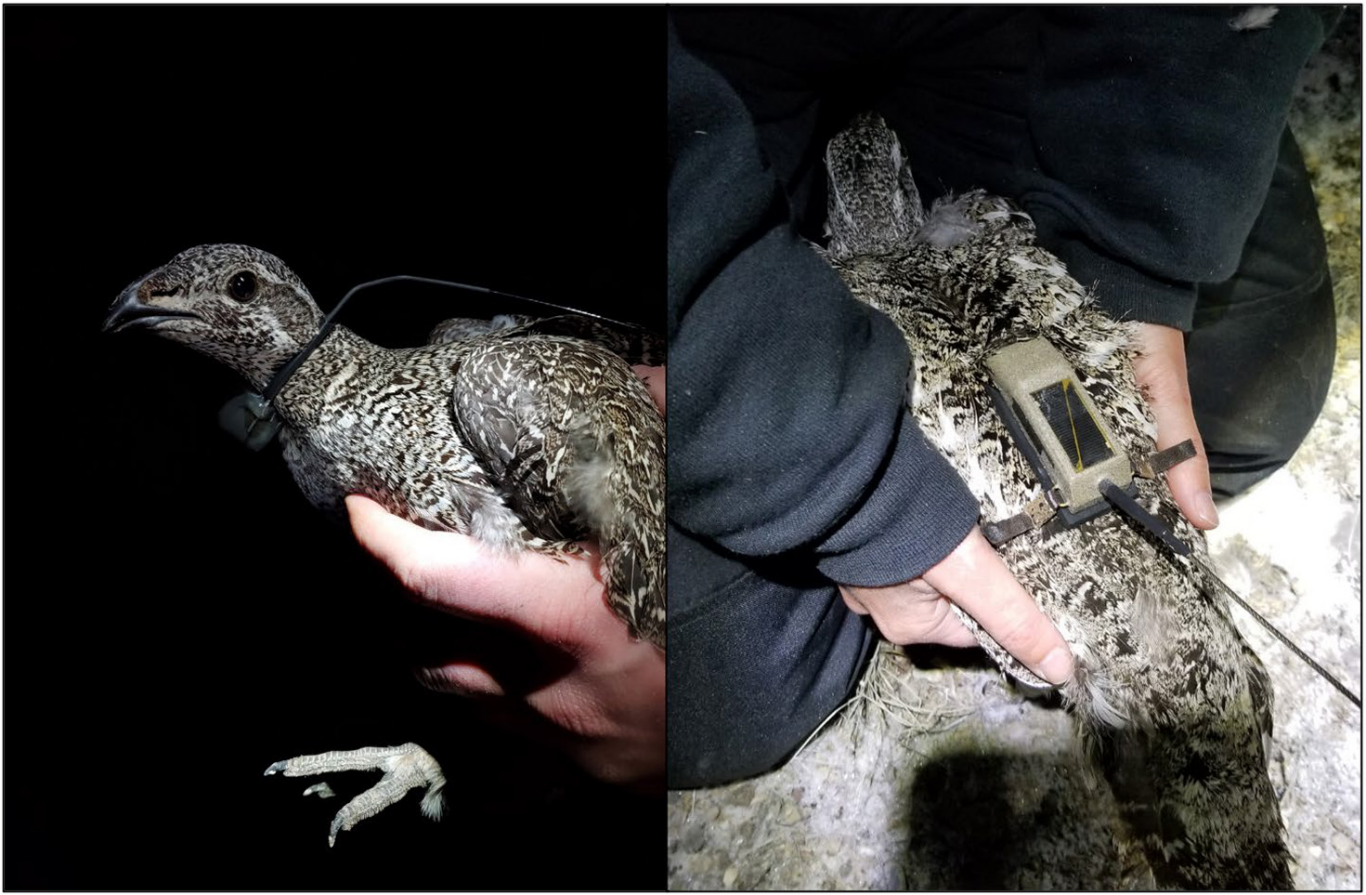
Pictures of necklace‐style very high frequency collar (left) and backpack‐style global positioning systems transmitters (right) attached to female greater sage‐grouse in the Pahsimeroi Valley of Idaho, USA.

We captured sage‐grouse hens at night during February 26–April 27 from 2016 to 2022 using spotlighting and hand netting (Gray, 1967; Wakkinen et al., 1992). For each bird, we recorded the capture location, body weight, and age (we used plumage characteristics to assign hens to adult or yearling age classes; Braun & Schroeder, 2015). We fit sage‐grouse with either a necklace‐style VHF radio transmitter (*n* = 199; hereafter VHF collars) or rump mounted, backpack‐style GPS platform terminal transmitter (*n* = 95; hereafter backpack GPS transmitter) and released hens at their locations of capture (Figure 2). Placement of backpack GPS transmitter began in 2017, after which VHF collars and backpack GPS transmitters were placed on individual grouse in a standardized and unbiased manner (e.g., deployment timing was similar); we typically had two trapping crews most nights, where individual field crews deployed a single transmitter type (either VHF or GPS) on a given trapping night and the type deployed changed among trap nights. VHF collars had a factory weight of 19–22 g and a deployed weight of 23.7–25.2 g (i.e., similar to the average deployed weight of 24.5 g and range of 24–25 g reported by Severson et al., 2019), with a pulse rate of 44 pulses per minute and pulse width of 21 milliseconds that switched to a mortality pulse rate of 90 pulses per minute after 4 h of inactivity (model A4050; Advanced Telemetry Systems, Isanti, MN). The backpack GPS transmitters were solar‐powered (Microwave Telemetry Inc., Columbia, MD), had a factory weight of 22 g and a deployed weight of approximately 32 g after including weight of the harness, and contained a UHF function for local field monitoring of hens marked with these transmitters. This contrasts with Severson et al. (2019) who deployed both 22‐ and 30‐g backpack GPS transmitters (but did not report the relative frequency of these weights) with an average deployed weight of 37.3 g (range = 35–40 g). Backpack GPS transmitters were tan colored with dimensions of 6.45 cm (length) × 2.34 cm (width) × 1.70 cm (height), and included a 17.78‐cm antenna that was hard nylon‐coated, flexible stranded marine‐grade steel and protruded from the rear of the transmitter at a 35° angle.

We used telemetry equipment to locate VHF‐marked sage‐grouse and attempted to locate hens every 2–3 days during nesting and brood rearing activities. If a VHF‐marked hen failed to hatch a brood and did not renest, then the frequency of survival monitoring was reduced to approximately once per week after the nesting period (approximately mid‐June). We monitored hens that moved to the periphery of the study area less frequently, approximately once per week. Monitoring for VHF‐marked hens was conducted >1 h after sunrise and >1 h before sunset to avoid disturbing female sage‐grouse on recess from nest incubation. We used a handheld Yagi antenna and VHF receiver to locate females via triangulation. For each VHF location, we recorded ≥3 directional azimuths from locations >50° apart with the observer attempting to stay 100–300 m away from the bird when recording azimuths. After a VHF‐marked female became localized by demonstrating consistent location for 2–3 consecutive visits, we approached the area cautiously to confirm if the bird was nesting and to pinpoint the nest location. We followed an explicit protocol for locating and monitoring nests that ensured minimum disturbance to nesting females. We approached females quietly to a distance of approximately 50 m and began circling the bird to identify the specific shrub or group of shrubs where the nest was located. We confirmed a nest was present by visual confirmation with binoculars (Aldridge & Brigham, 2002), or if we could not obtain a visual confirmation but thought we were close to the nest, we identified a shrub or cluster of shrubs from where the telemetry signal was emanating to avoid flushing the female while trying to locate the nest. We assumed the female was nesting within that cluster of shrubs if she was found in the same location on subsequent visits, even if a visual confirmation was never obtained. Moreover, we did not conduct these activities to pinpoint nest locations if nest predators (e.g., common ravens; *C. corax*) were observed in the area.

We established two monitoring points for checking each established nest that were marked with small rock cairns (Dahlgren et al., 2016) placed >100 m from the suspected nest site (Connelly et al., 1991). We monitored VHF‐marked females from these monitoring points every 2–3 days, where observers maintained a distance of >15 m from the suspected nest location, minimized the number of times they walked within 100 m of the nest site, and attempted to never flush females off of their nests. The two monitoring points were 90–150° apart (relative to the nest) and allowed us to confirm whether the female was still incubating with minimal disturbance from observers. We assumed a female was incubating a clutch of eggs if she was located at consistent bearings from the monitoring points. If bearings from the two monitoring points indicated the female was not near the nest during any monitoring visit, we walked into the area and searched the cluster of shrubs to locate the precise nest location and document its status. If the nest was active, we counted the eggs and quickly left the area. We determined the fate of each inactive nest (hatched or failed) based on the condition of eggshells (Connelly et al., 1991) and treated any nests thought to be abandoned as failed.

Monitoring methods for females marked with backpack GPS transmitters and their nests were slightly different than those for VHF‐marked hens. We programmed backpack GPS transmitters to collect six locations per day ($\bar{x}$ = 5.82, range = 1–7 locations per day because of failed location fixes or inadequate battery life at the time of a fix), and we assessed locations for potentially nesting females every 2–3 days using Google Earth to identify the suspected status of each bird (i.e., nesting or not). After a marked female began localizing her movements and a nest attempt was suspected, we completed a walk‐up nest search for each bird as soon as conditions allowed (typically within 1–2 days) to verify nesting status, following procedures described above. After nest locations were confirmed, we did not regularly visit the nests of females marked with backpack GPS transmitters during incubation, as we did for VHF‐marked females. Instead, we physically revisited nests only after location data indicated that incubation had ceased, and nest fate was assessed at this revisit as described above for VHF‐marked hens. During the intervening period between nest confirmation and the cessation of incubation, we assessed location data to determine whether the hen was still on the nest (i.e., was at the nest site), and therefore satellite fixes provided a remote nest check. Moreover, in the final 2 years of the study we did not physically verify nest locations to verify nesting status early in incubation, but instead relied entirely on remote nest checks from location and movement data until nest fate could be ascertained. Consequently, monitoring of nesting activities for sage‐grouse marked with backpack GPS transmitters included less disturbance of incubating females, as neither regular, physical nest checks nor rock cairn markers were required. Finally, we only monitored nesting activities of females marked with backpack GPS transmitters during 4 of the 7 study years (2019–2022) due to logistical constraints. Thus, in the nesting analyses described below, data from all 7 years contributed to estimating parameters for VHF‐marked birds, whereas the relative effects of backpack GPS transmitters were estimated using data from four of those years. Nonetheless, we conducted additional sensitivity analyses to ensure the unbalanced study design did not influence our conclusions about relative transmitter effects on nest survival (see Section 2.3).

We measured nesting propensity (i.e., whether or not a bird initiated a nest) and nest initiation dates for sage‐grouse marked with both VHF collars and backpack GPS transmitters. We calculated nesting propensity as the number of marked females that initiated at least one nesting attempt divided by the number of marked females that we tracked during the nesting period. However, a standard method for determining the denominator of this ratio is not well‐established, and therefore we used two approaches for characterizing the number of tracked females, and subsequently calculated two different measures of nesting propensity. For the first measure, we assumed a tracked bird was any female for which we either located a nest or obtained location data ≥1 day per week between the 14th and 23rd week of the year. For the second measure, we assumed a tracked bird was any female for which we either located a nest or obtained location data for >50% of the weeks between the 14th and 23rd week of the year (i.e., approximately the earliest and latest initiation times observed during the study). These two methods therefore represented conservative (i.e., yields fewer tracked females; method 1) and liberal (i.e., yields more tracked females; method 2) approaches to defining the denominator for estimating the fraction of hens that initiated a nest.

Finally, we estimated nest initiation dates for all marked hens. Sage‐grouse typically lay one egg every 1.5 days (Schroeder et al., 1999) and average clutch size is approximately seven eggs in Idaho (Connelly et al., 2011; Schroeder et al., 1999; Wakkinen, 1990). Therefore, we estimated the date of the first egg laid by subtracting 10.5 days (i.e., the time to lay the average clutch size of seven eggs with a laying interval of 1.5 days) from the estimated clutch completion date (i.e., the date incubation began). If we found evidence for >7 eggs in a particular nest, we used the number of eggs observed in our calculation of nest initiation date for that individual (i.e., we subtracted >10.5 days). However, we did not adjust nest initiation estimates if we detected eggshells suggesting the possibility of <7 eggs because our estimate of minimum clutch size was based on eggshell fragments located after the nest was no longer active, and consequently may have been smaller than the true clutch size.

### 2.3 Statistical analyses

We used the multistate model implemented in program MARK to estimate daily probability of mortality (i.e., the complement of daily survival probability) for sage‐grouse hens from March 1 to August 1 during 2016–2021 (Devineau et al., 2014; Lebreton & Pradel, 2002; White & Burnham, 1999). We used the multistate model to estimate mortality risk because our data were ragged telemetry data that in some cases included non‐detection (e.g., days with monitoring that attempted but failed to locate a given individual). We parameterized the model using two states, alive (A) and dead (D), where the state transition probability $\psi^{A\to D}$ represented the probability of transitioning from alive to dead in a given day (i.e., probability of mortality). Transitioning from dead to alive is obviously impossible, and therefore $\psi^{D\to A}$ was fixed at 0, and D represented an inescapable absorbing state ($\psi^{D\to D}=1$). We treated transmitter type (VHF vs. GPS) as a grouping variable for the analysis, which allowed us to estimate group‐specific mortality probabilities uniquely for each transmitter type ($\psi_{\mathrm{PTT}}^{A\to D}$ and $\psi_{\mathrm{VHF}}^{A\to D}$). The multistate model also allowed for failed detection when hens were searched for but not located. Thus, we modeled detection probability (*p*) for VHF‐marked hens because not all hens were detected on each day of attempted monitoring, whereas we fixed *p* = 1 for satellite GPS‐marked hens because failed detection was not an issue.

We created unique encounter histories with 154 encounter occasions (i.e., days, from March 1 to August 1) for each female during each breeding season (*n* = 353 encounter histories), and we modeled daily mortality probability from these data. Possible encounter observations included detected alive (A), detected dead (D), failed detection (0; VHF transmitters only), and no data (.). Observations of no data (.) were recorded, for example, if no monitoring was conducted for a given day or because of hens going missing or transmitter failure. In such cases, these days without observations do not contribute to the likelihood for estimating transition probabilities on that day, and hence this approach minimizes potential bias of survival estimation caused by censoring (Devineau et al., 2014). Because our primary goal was to assess transmitter differences in mortality risk for female sage‐grouse, we also retained a small number of birds in our analyses that died within 2 weeks of transmitter deployment (see Section 3), as disproportional differences in these numbers would provide further evidence for survival differences between the two transmitter types.

We wanted to estimate differences in daily mortality risk between transmitter types while also controlling for additional sources of variation in mortality (e.g., Severson et al., 2019). Therefore, we built an a priori model set that included effects of transmitter type on mortality, but also a binary variable for female age (adult vs. yearling), and temporal changes in mortality over the breeding season using linear, quadratic, and cubic time trends from March 1 to August 1. We considered the effects of 23 different combinations of those variables on $\psi^{A\to D}$, including additive effects and interactions between transmitter type, age, and time trends. We also considered two plausible models for the detection of VHF‐marked hens, including an intercept only model (*p*(.)) and a model with temporal heterogeneity in detectability (*p*(Time)). However, failed detection (0) was not observed for some years, and therefore Time was modeled as a categorical variable using three 2‐year intervals (2016–2017, 2018–2019, and 2020–2021) to ensure detection parameters were estimable. We therefore evaluated support for 46 candidate models to assess daily mortality risk for sage‐grouse (23 mortality models × 2 detection models). We note that preliminary analyses provided no evidence for annual heterogeneity in daily mortality probability, thus our analyses did not include annual effects and consequently estimated effects of transmitter type across all years (i.e., marginal effects averaged across all study years). We used the logit link function to model daily mortality and evaluated support for all candidate models using Akaike's information criterion adjusted for small sample sizes (AIC_c_) and normalized Akaike weights (*w*_*i*_; Burnham & Anderson, 2002). We also used model averaging to generate fitted values for partial effects plots and 95% confidence intervals on the real probability scale because of model selection uncertainty, and we present the estimated treatment effects for transmitter type on $\psi^{A\to D}$ for all competitive models (AIC_c_ ≤ 2).

We used the nest survival model in program MARK (Dinsmore et al., 2002) to estimate daily survival probability for sage‐grouse nests as a function of transmitter type and additional control covariates. We again treated transmitter type as a grouping variable for the analysis, which allowed us to estimate group‐specific daily nest survival probabilities uniquely for each transmitter type. We created an appropriately formatted input file that had the requisite data for modeling nest survival in MARK (day of nesting season that each nest was located, last day each nest was found intact, last day each nest was checked, and nest fate [success or failure]) for 255 unique nests (*n* = 164 VHF, *n* = 91 GPS; Table S1) observed over 7 years (2016–2022). Our primary goal was to assess transmitter effects on daily nest survival, yet we again wanted to control for additional sources of variation. Therefore, we built an a priori model set that included not only the effects of transmitter type but also individual covariates representing the study year (binary indicator variables for each year), ordinal day of nest initiation, and nest age (following Rotella et al., 2004). We considered 12 combinations of those variables on nest survival, including both linear and quadratic effects of ordinal day of nest initiation. We again used the logit link function and evaluated support using AIC_c_ and *w*_*i*_ values. To ensure our inferences about relative effects of transmitter type on nest survival were not corrupted by the unbalanced study design (Table S1), we conducted two additional sensitivity analyses: (1) refitting the top model but only including nests from the time period when both transmitter types were observed (2019–2022) and (2) refitting the model from #1 but also including a Year × Transmitter interaction to assess evidence for structured changes in transmitter effects among study years.

Finally, we used mixed‐effects generalized regression to test for changes in nesting behavior of backpack GPS‐marked hens relative to VHF‐marked hens. We used weighted binomial logistic regression with random intercepts for each study year, with weights determined by the number of females available to nest for each transmitter type and year, to test for differences in the proportion of nesting hens. This analysis used a binary variable to represent transmitter type and was replicated using both methods of measuring nesting propensity described above. Using only data from nesting females, we also tested for an effect of a binary indicator variable for transmitter type on the natural log transformed ordinal day of nest initiation using mixed‐effects Gaussian regression, again including random intercepts for each study year. We used the glmmTMB package (Brooks et al., 2017) in R version 4.3.0 (R Core Team, 2023) to fit all mixed‐effects models.

## 3 RESULTS

**FIGURE 3:**
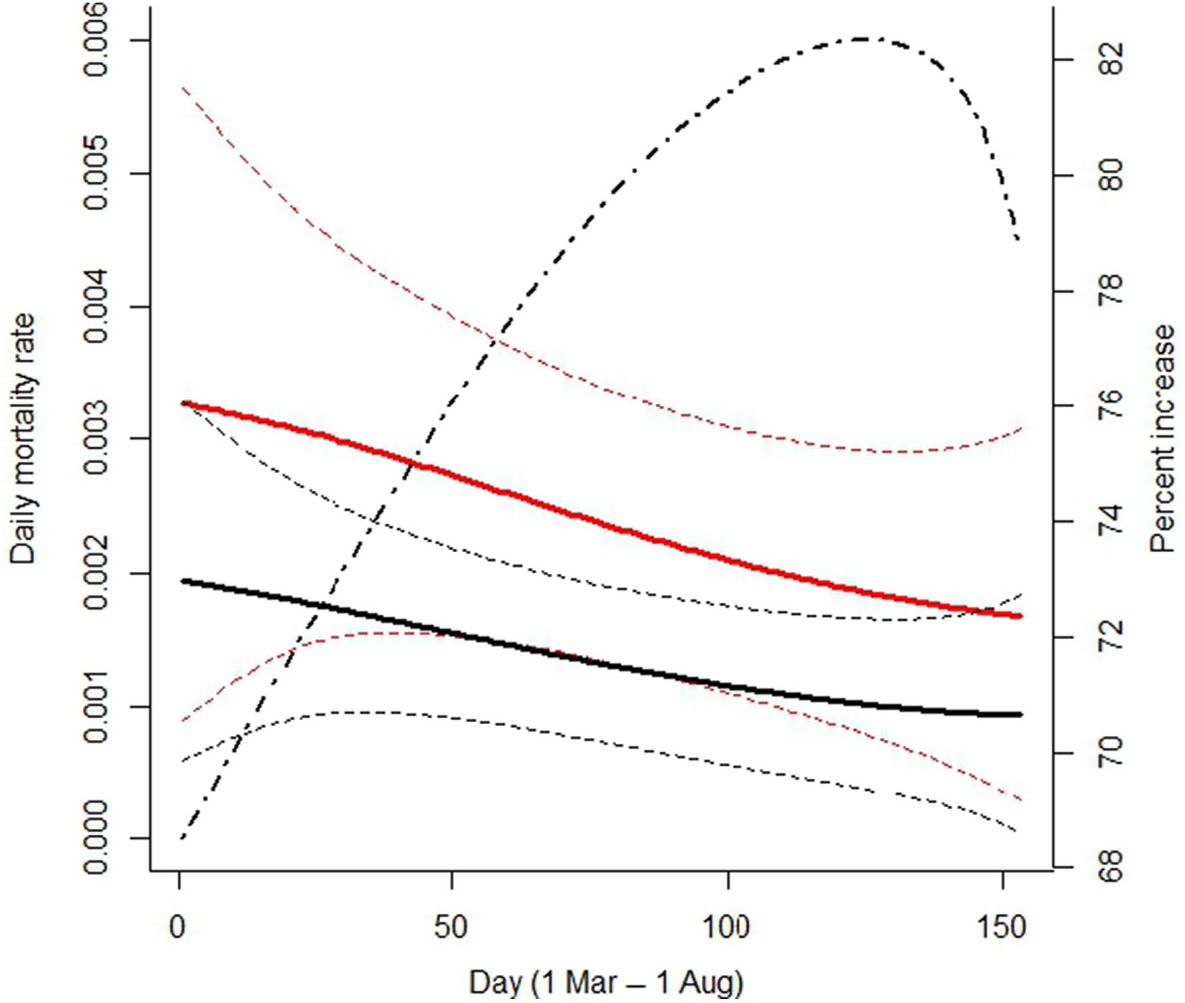
Model‐averaged probability of daily mortality for female greater sage‐grouse during the breeding season (March 1–August 1) in the Pahsimeroi Valley of central Idaho, 2016–2021. Daily mortality probability (left axis) is plotted as a function of transmitter type (backpack GPS = red, VHF collar = black) for each day of the breeding season, and 95% confidence intervals are represented by the dashed lines. Also shown is the percent increase in daily mortality probability (dot‐dash black line, right axis) for backpack GPS‐marked hens relative to VHF‐marked hens $\left(\frac{{\hat{\psi }}_{\mathrm{PTT}}^{A\to D}}{{\hat{\psi }}_{\mathrm{VHF}}^{A\to D}}\times 100\right)$ over the breeding season. Model averaging included models with an age covariate, and these plots were therefore generated assuming the adult segment of the population (age had minimal effects on mortality risk).

Our multistate model analysis demonstrated effects of transmitter type and a time trend over the breeding season on daily mortality probability for sage‐grouse hens, as well as temporal changes in detection probabilities for VHF‐marked hens over the study duration (Table 1 and Table S2; Figure 3). All top models (AIC_c_ ≤ 2) included a transmitter effect, whereas four of five top models included a time trend on survival over the breeding season. In contrast, hen age had little effect on survival. Consistent and statistically significant effects of transmitter type on daily mortality probability were observed (Table S2); 95% confidence intervals on the regression coefficients for transmitter effects excluded zero for all but one of the top models (i.e., all models except when a Transmitter × Trend interaction term was included), whereas the confidence intervals for the time trend excluded zero for two of four models containing the trend covariate (including the top model). Importantly, hens marked with backpack GPS transmitters had increased daily mortality risk relative to VHF‐marked hens, and model‐averaged daily mortality probability for hens marked with satellite GPS transmitters was 68%–82% higher than daily mortality probability for VHF‐marked hens during the spring–summer period (Figure 3). However, these increases were imposed on small values of daily mortality probability, and the point estimate of percent increase in cumulative survival probability from March 1 to August 1 (i.e., the product of $1-\psi^{A\to D}$ for each day) for VHF‐marked birds was approximately 17% (VHF‐marked hens = 81%, backpack GPS‐marked hens = 69%). Daily mortality probability was also highest early and declined over time during the spring–summer period. Nine marked hens died within 2 weeks of their first survival observation, and seven of these were hens marked with backpack GPS transmitters (i.e., 1.0% of *n* = 199 and 7.4% of *n* = 95 hens marked with VHF and backpack GPS transmitters, respectively). We found much weaker support for a Transmitter × Trend interaction term, a quadratic time trend, and age effects on daily mortality probability (Table 1), but coefficients for these terms were statistically non‐significant and their 95% confidence intervals included 0. Models without temporal changes in detection for VHF‐marked hens received no support (∆AIC_c_ ≥ 160).

**TABLE 1:** Model selection results for analyses testing the effects of transmitter type and control covariates on daily mortality probability for female greater sage‐grouse in the Pahsimeroi Valley of central Idaho, 2016–2021.

Model	∆AIC_c_	*w*_*i*_	*K*	−2LL
*ψ*(Transmitter + Trend), *p*(Time)	0.00	0.23	6	1839.6
*ψ*(Transmitter), *p*(Time)	1.14	0.13	5	1842.8
*ψ*(Transmitter + Trend + Transmitter × Trend), *p*(Time)	1.41	0.11	7	1839.0
*ψ*(Transmitter + Trend^2^), *p*(Time)	1.88	0.09	7	1839.5
*ψ*(Transmitter + Age + Trend), *p*(Time)	2.00	0.08	7	1839.6
*ψ*(Transmitter + Age), *p*(Time)	3.14	0.05	6	1842.8
*ψ*(.), *p*(Time)	3.14	0.05	4	1846.8
*ψ*(Transmitter + Trend^3^), *p*(Time)	3.19	0.05	8	1838.8
*ψ*(Trend), *p*(Time)	3.33	0.04	5	1845.0
*ψ*(Transmitter + Age + Trend^2^), *p*(Time)	3.88	0.03	8	1839.5
*ψ*(Transmitter + Age + Transmitter × Age), *p*(Time)	4.77	0.02	7	1842.4
*ψ*(Transmitter + Trend^2^ + Transmitter × Trend^2^), *p*(Time)	5.00	0.02	9	1838.6

*Note*: The multistate model parameter *ψ* represents the daily mortality probability (i.e., probability of transitioning from alive state to dead state), whereas *p* represents the conditional detection probability for VHF‐marked hens (*p* was set to 1 for backpack GPS‐marked hens). Transmitter types were VHF collars and backpack GPS, Trend represents a linear time trend over the breeding season (March 1–August 1), Age is a binary indicator variable representing adults or yearlings, and Time is a categorical variable with 2‐year periods pooled into categories (i.e., 2016–2017, 2018–2019, and 2020–2021). Models were ranked using Akaike's information criterion corrected for small sample sizes (AIC_c_), and only models with ∆AIC_c_ ≤5 are displayed (cumulative model weight of 0.9 for models displayed). Also shown are the Akaike model weights (*w*_*i*_), the number of model parameters estimated (*K*), and −2 × log likelihood (−2LL) calculated for each model.

**FIGURE 4:**
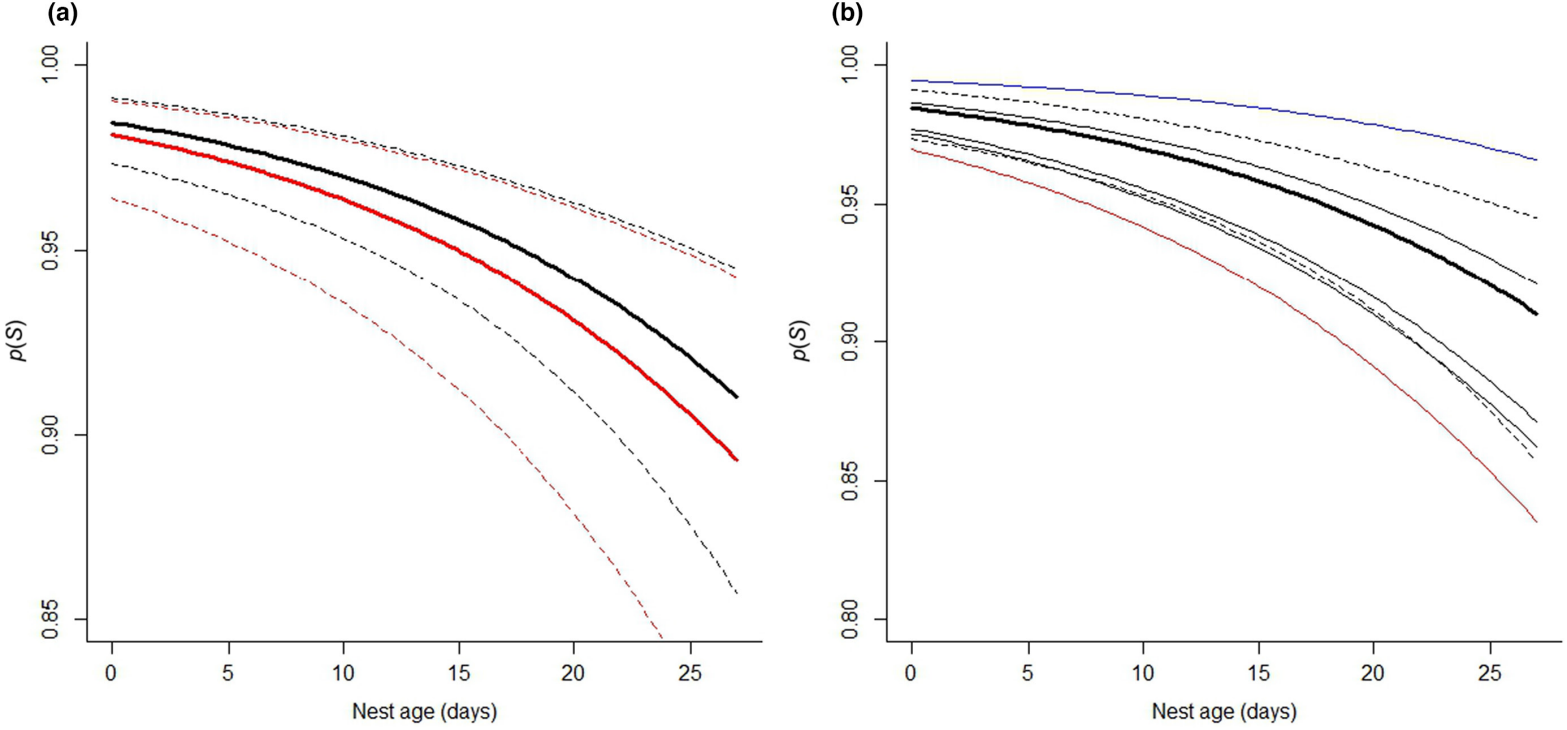
Marginal effect plots for nest age and transmitter type (VHF collar = black, backpack GPS = red) effects on probability of daily nest survival (*p*(*S*)) from the top nest survival model for greater sage‐grouse in the Pahsimeroi Valley of central Idaho, 2016–2022. Panel a plots nest age effects for both transmitter types (for the intercept year, 2016), whereas panel b shows annual heterogeneity of nest survival (VHF‐marked birds only). Specifically, in panel b, the thick black line represents the intercept year, the thin black lines are years not statistically different from 2016, the red line is the year significantly lower than 2016 (2017), and the blue line is the year with significantly higher nest survival than 2016 (2020). Dashed lines represent 95% confidence intervals for the intercetp year (thick black line) and both plots were generated at the median observed value for the ordinal day of nest initiation.

**FIGURE 5:**
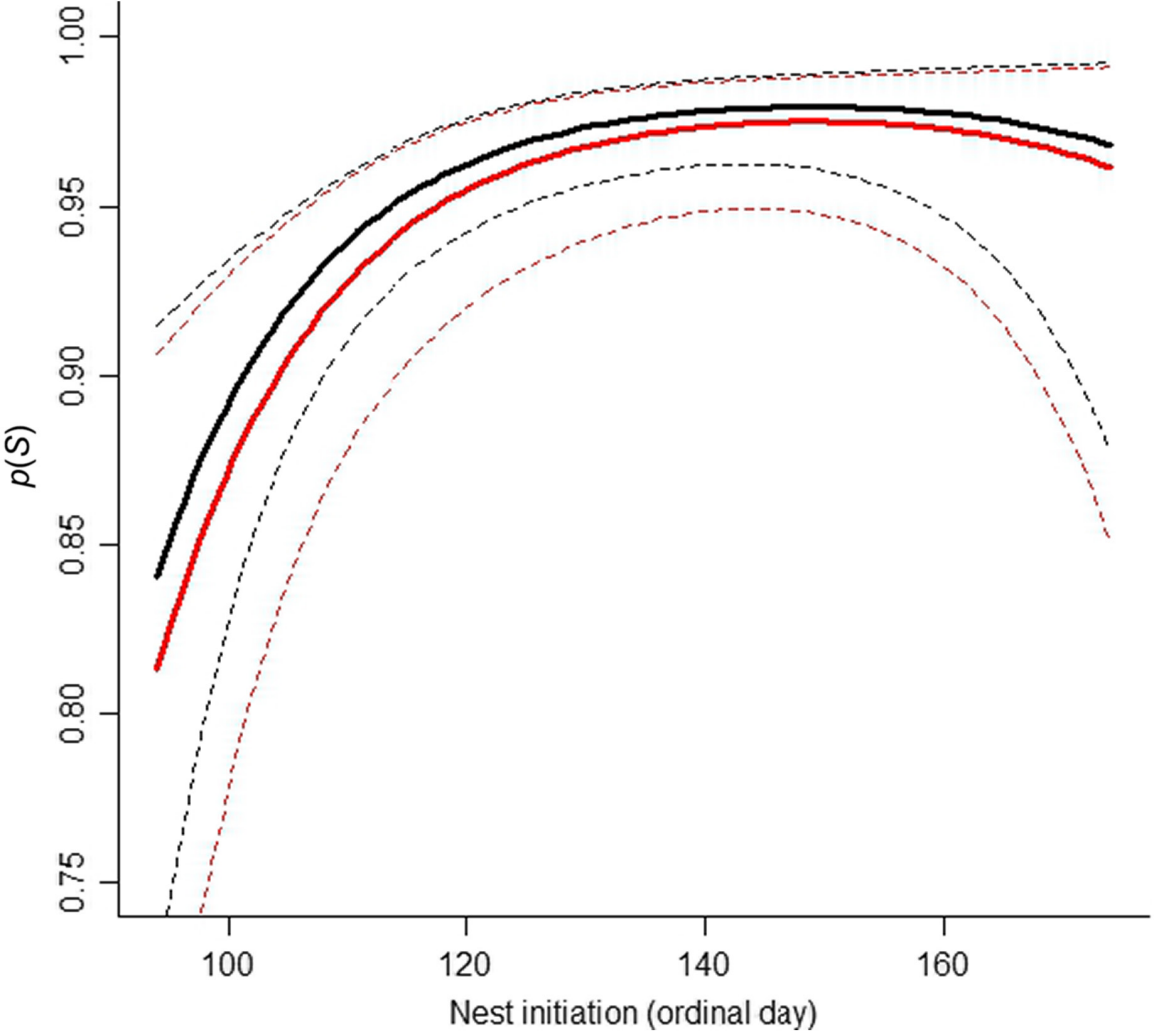
Partial effect of nest initiation day and transmitter type (VHF collar = black, backpack GPS = red) on probability of daily nest survival (*p*(S)) from the top nest survival model for greater sage‐grouse in the Pahsimeroi Valley of central Idaho, 2016–2022. Dashed lines represent 95% confidence intervals, and the plot was generated for a nest age of 14 days (i.e., midpoint of incubation) and for the model intercept year (2016).

The top nest survival model included nest age, ordinal day of nest initiation, year, and transmitter effects, and there was no model selection uncertainty (∆AIC_c_ ≥ 20 to second best model; Table 2). The transmitter effect on nest survival was both biologically and statistically non‐significant; however, the effects of nest age, initiation date, and year were both biologically and statistically significant (Table 3; Figures 4 and 5). Daily nest survival declined strongly as nests increased in age (Figure 4). For example, daily nest survival probability decreased by approximately 9% from age 1 to 27 days after initiation (for year = 2016 and at the median value of nest initiation date; Figure 4). We also observed heterogeneity in the probability of daily nest survival among years, where survival was the lowest in 2017 and the highest in 2020 (Figure 4). Moreover, daily nest survival was the lowest for nests initiated early in the season (Figure 5). For example, daily nest survival increased by approximately 18% from April 3 (Day 94) to April 29 (Day 120) and remained consistent for nests initiated thereafter (Figure 5). Sensitivity analyses that used only data from 2019 to 2022 showed that: (1) main effects and inferences from the top model were little changed (as compared to the full data set; Table 3; Table S3), and (2) statistically significant interactions between transmitter type and study year were observed but produced no biologically meaningful pattern regarding relative effects of backpack GPS transmitters on daily nest survival (Table S4). More specifically, daily nest survival appeared lower for backpack GPS transmitters in 2019, which was not otherwise a year with lower nest survival (Table 3; Table S3), but was no different between transmitter types in any other year (Table S4). Finally, we found no evidence that transmitter type affected nesting propensity (method 1: *β* = 0.95, 95% CI = −0.67 to 2.57; method 2: *β* = −0.63, 95% CI = −1.56 to 0.31; backpack GPS transmitters as intercept) or the date of nest initiation (*β* = −0.01, 95% CI = −0.04 to 0.01; backpack GPS transmitters as intercept).

**TABLE 2:** Model selection results from nest survival analyses for female greater sage‐grouse in the Pahsimeroi Valley of central Idaho, 2016–2022.

Model	∆AIC_c_	*w*_*i*_	*K*	−2LL
*S*(Transmitter + Year + Age + Initiation)	0.00	1.00	11	1028.86
*S*(Transmitter + Year + Age)	20.51	0.00	9	1053.39
*S*(Transmitter + Age + Initiation)	23.64	0.00	5	1064.56
*S*(Age)	29.94	0.00	2	1076.87
*S*(Transmitter + Age)	31.84	0.00	3	1076.77
*S*(Transmitter + Year + Initiation^2^)	34.42	0.00	10	1065.29
*S*(Initiation^2^)	35.76	0.00	3	1080.68
*S*(Transmitter + Initiation^2^)	37.61	0.00	4	1080.53
*S*(.)	50.04	0.00	1	1098.97
*S*(Transmitter)	51.85	0.00	2	1098.78
*S*(Year)	53.12	0.00	7	1090.02
*S*(Transmitter + Year)	55.01	0.00	8	1089.90

*Note*: The nest survival model parameter *S* represents the daily survival probability for individual nests as a function of transmitter type (VHF collar or backpack GPS), nest age (Age), ordinal day of nest initiation (Initiation), and a categorical variable representing study years (2016, 2017, 2018, 2019, 2020, 2021, 2022). Models were ranked using Akaike's information criterion corrected for small sample sizes (AIC_c_), and all candidate models are displayed. Also shown are the Akaike model weights (*w*_*i*_), the number of model parameters estimated (*K*), and −2 × log likelihood (−2LL) calculated for each model.

**TABLE 3:** Parameter estimates (β) and 95% confidence intervals (CIs) from the top nest survival model for greater sage‐grouse in the Pahsimeroi Valley of central Idaho, 2016–2022.

Parameter	*β*	CI
Intercept (VHF and Year = 2016)	−11.24	−21.00 to −1.48
Transmitter (GPS = 1)	−0.19	−0.59 to 0.20
Year = 2017	−0.69	−1.35 to −0.03
Year = 2018	−0.48	−1.12 to 0.16
Year = 2019	−0.01	−0.61 to 0.60
Year = 2020	1.03	0.40 to 1.66
Year = 2021	0.14	−0.54 to 0.82
Year = 2022	−0.40	−1.03 to 0.22
Nest age	−0.07	−0.09 to −0.05
Initiation day	0.22	0.06 to 0.37
Initiation day^2^	−0.001	−0.001 to 0.00

*Note*: The top model included a binary indicator variable for transmitter type (VHF collar or backpack GPS), a categorical variable representing study years (2016, 2017, 2018, 2019, 2020, 2021, and 2022), and continuous covariates representing nest age and ordinal day of nest initiation.

## 4 DISCUSSION

This work corroborates two recent studies suggesting reduced survival for sage‐grouse marked with backpack GPS transmitters in the southwest portion of their range (Foster et al., 2018; Severson et al., 2019), and is the first to evaluate effects of backpack GPS transmitters on nesting propensity, timing of nest initiation, and nest survival. Backpack GPS transmitters reduced survival of sage‐grouse hens but did not change the likelihood or timing of nest initiation relative to VHF collars, nor did they consistently affect nest success. Instead, propensity to nest was high and timing of initiation was indistinguishable between hens marked with backpack GPS transmitters and those marked with VHF collars. Moreover, daily nest survival varied across years and with both timing of nest initiation and nest age, and a post hoc sensitivity analysis showed that any measurable differences in nest survival between transmitter types were inconsistent over time. Therefore, our results provide additional context to recent concerns about transmitter effects on sage‐grouse (e.g., Foster et al., 2018; Severson et al., 2019) and suggest that while trade‐offs between transmitter types and survival should be expected, there was little evidence for similar trade‐offs between transmitter type and nesting behavior or success.

Backpack GPS transmitters reduced survival of sage‐grouse in the Great Basin (Severson et al., 2019), and we provide further evidence for reduced survival from the northern portion of the sage‐grouse range. Sage‐grouse hens in California and Nevada that were marked with VHF collars had seasonal survival rate estimates 8%–19% higher than hens marked with backpack GPS transmitters, based on posterior median point estimates of survival for spring, summer, fall, and winter, respectively (Severson et al., 2019). Backpack GPS transmitters reduced annual survival of female sage‐grouse in Oregon by approximately 5%, relative to VHF collars (Foster et al., 2018), but this difference was not statistically significant. Our results indicate that the probability of surviving the period from March 1 to August 1 was approximately 17% higher for sage‐grouse hens marked with VHF collars, relative to those marked with backpack GPS transmitters. Our seasonal period does not align perfectly with the time periods described by Severson et al. (2019), yet we both reported an increase in survival from spring to summer. Thus, our results closely corroborate and validate those reported from the southern Great Basin and provide additional evidence that backpack GPS transmitters attached via rump‐mounted harnesses reduce survival of sage‐grouse hens.

The underlying mechanisms regarding why sage‐grouse hens marked with backpack GPS transmitters have lower survival remain unclear. Backpack transmitters and VHF collars differ in several ways, including the deployed weight (lighter for VHF), attachment method (backpack vs. necklace), positioning (neck vs. rump), and the presence of reflective solar panels (GPS only), all of which have been directly or anecdotally implicated as affecting the survival of Galliformes (Amstrup, 1980; Burger et al., 1991; Severson et al., 2019; Small & Rusch, 1985; Warner & Etter, 1983). Yet, these attribute differences were statistically confounded in our study and those conducted previously, and no studies have designed experiments to tease apart the precise causes of reduced survival for birds marked with backpack GPS transmitters.

Our results nonetheless provide some clues as to hypothesized causes of reduced survival. First, the deployed weight of our backpack GPS transmitters was approximately 10% lighter than the average weight reported by Severson et al. (2019), yet our survival increase from backpack GPS transmitters to VHF collars (17%) was near the high end of the seasonal range they reported (8%–19%). Also, if increased weight resulted in decreased physical condition for hens carrying backpack GPS transmitters, we expected that decreased physical condition would manifest itself through reduced nesting propensity, nest initiation date, or nest survival (e.g., because of increased foraging off nest), which we did not find. Together these results suggest that weight differences may not be the ultimate cause of reduced survival for hens marked with backpack GPS transmitters. Moreover, if the reflective properties of solar panels affixed to satellite GPS transmitters attract predators (e.g., as suggested by Burger et al., 1991), this would also predict reductions to nest survival. This was not supported by our findings, nor was the prediction of increased nest survival associated with decreased observer disturbance for hens marked with backpack GPS units. These results therefore lead us to conclude that the backpack harness or its attachment methods (e.g., Kircher et al., 2020), the transmitter positioning on the rump of the bird, or both, may be driving the observed survival differences between VHF collars and backpack GPS transmitters. Nonetheless, the causal mechanisms driving demographic changes between sage‐grouse marked with VHF collars and backpack GPS transmitters need to be identified via carefully designed experiments that remove the confounding of attributes related to transmitter design and deployment.

Although backpack GPS transmitters reduced survival of sage‐grouse hens relative to VHF‐marked hens, we found little evidence that transmitter type consistently affected nesting propensity, timing of nest initiation, or nest survival. Sage‐grouse nesting propensity is generally high (Connelly et al., 2011) and we found no evidence that backpack GPS transmitters reduce this strong tendency to initiate a nest. Timing of nest initiation affected nest survival, but transmitter type did not consistently affect either of these metrics. Thus, we conclude that differences in transmitter design and deployment did not meaningfully impact important attributes of nesting that relate to per capita productivity and contribute to population growth (Taylor et al., 2012), but also recognize that nest survival varies strongly across years and the effects of transmitter type on multiple facets of breeding behavior remain unstudied (e.g., habitat use, movements, etc.). Daily nest survival varied among years, was lower for nests initiated early in the breeding season, and declined over time as incubation progressed. These results corroborate past studies of sage‐grouse nesting ecology conducted in other parts of their range (e.g., Kolada et al., 2009; Moynahan et al., 2007). Similarly, daily nest survival changed with respect to each of these covariates (annual change, timing of nest initiation, nest age) for Gunnison sage‐grouse (*Centrocercus minimus*) in Colorado (Davis et al., 2015). Consequently, the relationships between our control covariates and nest survival provide greater confidence in our results given their consistency with past studies.

Our study contributes valuable information to researchers seeking to understand the trade‐offs of different transmitter options for sage‐grouse. While the lack of a consistent effect of backpack GPS transmitters on nesting sage‐grouse should be welcome, the effects of backpack GPS transmitters on hen survival remain, as do the practical and ethical implications for research (see broader discussion by Severson et al., 2019). Backpack GPS transmitters provide improved temporal and spatial resolution for understanding many aspects of sage‐grouse behavior and demography given a fixed amount of effort. Yet, at least under current designs, the survival implications for sage‐grouse marked with backpack GPS transmitters have been replicated with surprising consistency using large samples of marked grouse (Severson et al., 2019: 873 birds marked over 6 years; this study: 294 birds marked over 6 years) over diverse regions across southern (California and Nevada) and northern (Idaho) parts of the sage‐grouse range. Further technological advancements could reduce the weight of backpack GPS transmitters and facilitate necklace‐style deployment designs for sage‐grouse. At present, however, researchers will need to carefully balance the information needs and research goals of studies on the behavior and demography of sage‐grouse against the practical reality that acquiring the volume of data generated by backpack GPS transmitters will require considerably more field effort if attempting to do so using VHF telemetry. Some study questions may require such high‐resolution and high‐volume data (e.g., assessing fine‐scale movement and behavior), whereas for other questions (e.g., estimating demographic parameters) or for studies conducted on small or declining populations, these value judgments may change. Regardless, our results replicate important findings regarding the effects of backpack GPS transmitters on sage‐grouse survival, but also provide solace in that these transmitters do not appear to affect important components of fecundity.

## AUTHOR CONTRIBUTIONS

**Bryan S. Stevens:** Conceptualization (equal); data curation (supporting); formal analysis (lead); methodology (supporting); writing – original draft (lead); writing – review and editing (equal). **Courtney J. Conway:** Conceptualization (equal); data curation (lead); formal analysis (supporting); funding acquisition (lead); methodology (lead); project administration (lead); writing – review and editing (equal). **Cody A. Tisdale:** Conceptualization (supporting); data curation (lead); formal analysis (supporting); methodology (supporting); project administration (supporting); writing – review and editing (equal). **Kylie N. Denny:** Conceptualization (supporting); data curation (lead); formal analysis (supporting); methodology (supporting); writing – review and editing (equal). **Andrew Meyers:** Conceptualization (supporting); data curation (supporting); methodology (supporting); project administration (supporting); writing – review and editing (equal). **Paul Makela:** Conceptualization (supporting); funding acquisition (supporting); methodology (supporting); project administration (supporting); writing – review and editing (equal).

## CONFLICT OF INTEREST STATEMENT

The authors declare that they have no known competing financial interests or personal relationships that could have appeared to influence the work reported in this paper.

## Supporting information


Appendix S1.

## Data Availability

The data files and R scripts analyzed to support this study are archived on Dryad (https://doi.org/10.5061/dryad.573n5tbf3).
