# Supplementary material for: Backpack satellite transmitters reduce survival but not nesting propensity or success of greater sage‐grouse
Source: Ecol Evol. 2023 Dec 18;13(12):e10820. doi: 10.1002/ece3.10820 (PMC10726286; doi:10.1002/ece3.10820)
Supplement: Supplementary file 1 — Appendix S1. [file ECE3-13-e10820-s001.docx]

**Appendix S1:** **Additional supporting results for assessing impacts of satellite GPS transmitters on survival and breeding of female sage grouse in the Pahsimeroi Valley of Idaho, USA.**

Table S1. Sample sizes for nests that were used to assess relative effects of transmitter types (VHF collar and backpack GPS) on greater sage-grouse nest survival from the Pahsimeroi Valley of central Idaho, 2016-2021.

| Year | VHF | GPS |
| --- | --- | --- |
| 2016 | 32 | 0 |
| 2017 | 25 | 0 |
| 2018 | 26 | 0 |
| 2019 | 23 | 20 |
| 2020 | 23 | 25 |
| 2021 | 17 | 20 |
| 2022 | 18 | 26 |
| Total | 164 | 91 |

Table S2. Robustness of conclusions about the effects of transmitter type on daily mortality probability for female greater sage-grouse in the Pahsimeroi Valley of central Idaho, 2016-2021. The multistate model parameter ψ represents the daily mortality probability (i.e., probability of transitioning from alive state to dead state), whereas *p* represents the conditional detection probability for VHF-marked hens (*p* was set to 1 for backpack GPS-marked hens). Transmitter types were VHF collars (0) and backpack GPS transmitters (1), Trend represents a linear time trend over the breeding season (1 Mar – 1 Aug), Age is a binary indicator variable representing adults or yearlings, and Time is a categorical variable with 2-year periods pooled into categories (i.e., 2016-2017, 2018-2019, 2020-2021). Presented here are the coefficients estimating effects of backpack GPS transmitters on daily mortality (β_transmitter_) and 95% confidence intervals (CI) for those estimates for each of the most competitive models (∆AIC_c_ ≤ 2).

| Model | ∆AIC_c_ | β_transmitter_ | CI |
| --- | --- | --- | --- |
| ψ(Transmitter + Trend), p(Time) | 0.00 | 0.69 | 0.11–1.27 |
| ψ(Transmitter), p(Time) | 1.14 | 0.59 | 0.02–1.16 |
| ψ(Transmitter + Trend + Transmitter*Trend), p(Time) | 1.41 | 0.30 | -0.89–1.45 |
| ψ(Transmitter + Trend^2^), p(Time) | 1.88 | 0.70 | 0.12–1.29 |
| ψ(Transmitter + Age + Trend), p(Time) | 2.00 | 0.69 | 0.10–1.27 |

Table S3. Parameter estimates ($\beta$) and 95% confidence intervals (CI) from the top nest survival model for greater sage-grouse in the Pahsimeroi Valley of central Idaho, re-fit using only data from 2019-2022. The top model included a binary indicator variable for transmitter type (VHF collar or backpack GPS), a categorical variable for year (2019, 2020, 2021, and 2022), and continuous covariates representing nest age and ordinal day of nest initiation.

| Parameter | β | CI |
| --- | --- | --- |
| Intercept (VHF and Year = 2019) | -6.97 | -19.59–5.66 |
| Transmitter (GPS = 1) | -0.19 | -0.59–0.21 |
| Year = 2020 | 0.77 | 0.15–1.39 |
| Year = 2021 | 0.11 | -0.47–0.70 |
| Year = 2022 | -0.44 | -0.99–0.11 |
| Nest age | -0.05 | -0.08– -0.03 |
| Initiation day | 0.15 | -0.04–0.35 |
| Initiation day^2^ | -0.0005 | -0.001–0.0002 |

Table S4. Estimated daily nest survival probabilities (DSR) and their 95% confidence intervals (CI) from the top nest survival model for greater sage-grouse in the Pahsimeroi Valley of central Idaho, re-fit to include an interaction between study year and transmitter type and using only data from 2019-2022. Transmitter types were VHF collars and backpack GPS transmitters, and daily survival probability estimates were generated at the median ordinal day of nest initiation (day 119) and for a nest of age 14 days. This table shows that despite significant Year×Transmitter effects, there was no consistent pattern of nest survival differences observed between VHF collars and backpack GPS transmitters.

|  | 2019 | | 2020 | | 2021 | | 2022 | |
| --- | --- | --- | --- | --- | --- | --- | --- | --- |
| Transmitter | DSR | CI | DSR | CI | DSR | CI | DSR | CI |
| VHF | 0.975 | 0.956–0.986 | 0.984 | 0.969–0.992 | 0.949 | 0.910–0.972 | 0.921 | 0.870–0.953 |
| GPS | 0.928 | 0.886–0.956 | 0.977 | 0.960–0.987 | 0.970 | 0.945–0.984 | 0.943 | 0.908–0.964 |
